# Carboxymethyl Cellulose-Grafted Mesoporous Silica Hybrid Nanogels for Enhanced Cellular Uptake and Release of Curcumin

**DOI:** 10.3390/gels3010008

**Published:** 2017-02-22

**Authors:** Neha Tiwari, Laxman Nawale, Dhiman Sarkar, Manohar V. Badiger

**Affiliations:** 1 Polymer Science and Engineering Division, CSIR-National Chemical Laboratory, Pune 411008, India; n.tiwari@ncl.res.in; 2 Academy of Scientific & Innovative Research, CSIR-NCL Campus, Pune 411008, India; d.sarkar@ncl.res.in; 3 Combichem Bioresource Centre, Organic Chemistry Division, CSIR-National Chemical Laboratory, Pune 411008, India; lu.nawale@ncl.res.in

**Keywords:** curcumin, mesoporous silica nanoparticles, carboxymethyl cellulose, drug delivery

## Abstract

Mesoporous silica nanoparticles (MSNs) with ordered pore structure have been synthesized and used as carriers for the anticancer drug curcumin. MSNs were functionalized with amine groups and further attached with carboxymethyl cellulose (CMC) using 1-ethyl-3-(3-dimethylaminopropyl)-carbodiimide (EDC) coupling chemistry, which increased the hydrophilicity and biocompatibility of MSNs. The functionalized MSNs (MSN-NH_2_ and MSN-CMC) were characterized using Scanning Electron Microscopy (SEM), Transmission Electron Microscopy (TEM), Dynamic Light Scattering (DLS), N_2_ adsorption, X-Ray Diffraction (XRD), Thermo Gravimetric Analysis (TGA) and Fourier Transform Infrared Spectroscopy (FT-IR). The in vitro release of curcumin from the –NH_2_ and CMC functionalized MSNs (MSN-cur-NH_2_ and MSN-cur-CMC) was performed in 0.5% aqueous solution of sodium lauryl sulphate (SLS). The effect of CMC functionalization of MSNs towards cellular uptake was studied in the human breast cancer cell line MDA-MB-231 and was compared with that of MSN-NH_2_ and free curcumin (cur). Both MSN-NH_2_ and MSN-CMC showed good biocompatibility with the breast cancer cell line. The MTT assay study revealed that curcumin-loaded MSN-cur-CMC showed better uptake as compared to curcumin-loaded MSN-cur-NH_2_. Free curcumin was used as a control and was shown to have much less internalization as compared to the curcumin-loaded functionalized MSNs due to poor bioavailability. Fluorescence microscopy was used to localize the fluorescent drug curcumin inside the cells. The work demonstrates that CMC-functionalized MSNs can be used as potential carriers for loading and release of hydrophobic drugs that otherwise cannot be used effectively in their free form for cancer therapy.

## 1. Introduction

Cancer has become a major concern worldwide as most of the drugs effective in its treatment are hydrophobic in nature and thus have less bioavailability at the required site. Chemotherapy, although clinically accepted for the treatment of cancer, has major side effects linked to the poor specificity of chemotherapeutic, which leads to the death of normal cells along with the cancer cells [1]. Developing controlled-release technology to avoid premature drug release with targeted drug delivery may provide more efficient and less harmful solution compared to conventional chemotherapy. In this context, nanotechnology has emerged as one of the major areas of research for drug delivery towards cancer treatment to overcome the above mentioned problems [2,3]. Various efforts have been made in recent years towards the synthesis of nanocarriers for drug delivery and preventing the premature release of drugs before reaching the targeted site. The nanocarriers for drug delivery must possess certain desired properties like biocompatibility, bioavailability, improved circulation half time, chemical stability in in-vivo conditions, inertness towards enzymatic degradation, tissue specificity and controlled drug release. Although various nanocarriers like liposomes, micelles, capsules, dendrimers, carbon nanotubes [4] etc. have been studied, they lack stability in in-vivo conditions and are prone to various enzymatic degradation before reaching the targeted site and results in premature drug release [4]. Recently, inorganic mesoporous silica nanoparticles (MSNs) have attracted increasing attention in bio-medical applications due to their stability in in-vivo conditions, biocompatibility and ease of synthesis [5,6]. The particle and pore size of MSNs can be controlled depending on their applications which make them highly versatile in nature [7,8,9]. With large surface area, porosity and tuneable pore size, control over the functionalization of surface, ordered mesopores, biocompatibility and stability, MSNs have great advantages over other nanocarriers [9]. The template synthesis is one of the strategy to make mesopores in silica particles which has proven to be a potential route to achieve desired nano architectures [10]. The ordered porous structure in MSNs is important in wide range of applications such as catalysis, adsorption, optics, photochemistry etc. [11]. The control over the dimensions, morphologies, composition and porosity of MSNs has been exploited for synthesizing integrated nanocrystals (INCs) in catalytic applications [12]. MSNs have been successfully explored for various biological applications like drug delivery [13,14,15], biosensors [16], gene transfection [17,18] etc. Particularly, porous MSNs have become important since their porosity can be successfully utilized for the encapsulation of hydrophobic drugs [19,20] and other bio-molecules. The highly porous structure of MSNs facilitates loading large amounts of drugs so that minimum amount of the carrier is sufficient for drug delivery at the cancer site by enhanced cell permeability (EPR) without any potential side effects. Well-established theory postulates that MSNs tend to accumulate at the cancer site more effectively as compared to normal cells [21,22]. Further, both the outer surface and the pores of MSNs can be selectively functionalized with organic moieties to enhance their biocompatibility and increased circulation time in blood [23]. The functionalization of MSNs can also help in preventing the drug or other bio-molecules present inside the porous structure from enzymatic degradation before reaching the targeted site. Surface functionalization could be achieved by modification of the surface of MSNs with organic moieties that are biocompatible.

A literature review reveals that surface modifications have been carried out using both synthetic molecules like polyethylene glycol [24], folic acid [25], β-cyclodextrin [26] and polyacrylic acid [27] as well as naturally occurring molecules like amino acids (poly-l-lysine and poly-l-arginine) [17] which are biocompatible with the cell environment. Polysaccharides are another class of naturally occurring polymers with inherent biocompatible and biodegradable properties which can be used to enhance the biocompatibility of MSNs without any potential side effects. For example, chitosan [28,29], hyaluronic acid [30], mannose [31] and alginate [32] are a few of the naturally occurring polysaccharides that have been studied extensively for encapsulation of hydrophobic drug molecules and enhanced permeation of hybrid silica particles inside the tumour cells. Other potential polysaccharides like carboxymethyl cellulose (CMC), carboxymethyl tamarind (CMT) and guar gum (GG) in combination with MSNs are yet to be fully explored for drug delivery applications. Another advantage of the polysaccharides is their degradability by enzymes, by which they can be coated onto drug loaded MSNs and the drug can be released by polysaccharide degradation.

In the present work, we have prepared the functionalized MSNs with a polysaccharide, namely carboxy methyl cellulose (CMC) using an EDC coupling reaction between amine functionalized MSNs (MSN-NH_2_) and the carboxylic groups of CMC. The pores of MSNs were incubated with the hydrophobic drug curcumin, which has both anticancer and antibacterial activity. It was observed that CMC modified MSNs helped in the enhanced permeation of MSNs inside the tumour cells. An MTT assay of breast cancer cell line, MDA-MB-231 revealed that curcumin loaded and CMC coated MSNs (MSN-cur-CMC) showed better cell inhibition compared to the curcumin loaded MSN-cur-NH_2_ or free curcumin. Apoptosis studies performed on cancer cell line MDA-MB-231 indicated that MSN-cur-CMC shows apoptosis at a lower GI_50_ concentration compared to that of MSN-cur-NH_2_. Therefore, CMC grafted MSNs show great promise in enhanced internalization of drug molecules inside the cancer cell lines.

## 2. Results and Discussion

### 2.1. Functionalization and Characterization of Mesoporous Silica Nanoparticles (MSNs)

**Scheme 1:**
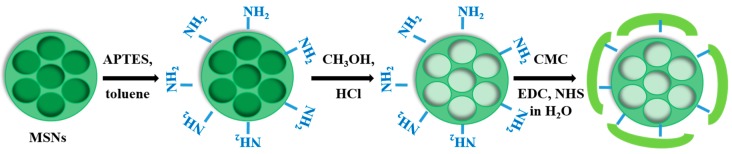
Synthesis of carboxymethyl cellulose (CMC)-grafted mesoporous silica nanoparticles (MSNs).

Mesoporous silica nanoparticles (MSNs) were synthesized according to the previous reports [14,33]. The synthetic route of MSNs and its functionalization with amine moieties and carboxymethyl cellulose is given in Scheme 1. Briefly, the synthesis of MSNs was carried out by sol-gel method using cetyltrialkyl amonium bromide (CTAB) as a structure-directing agent and tetraethyl orthosilicate (TEOS) as a silica precursor [17,34]. Functionalization of the outer surface of MSNs with amine moieties was performed using aminopropyl triethoxysilane (APTES). The amino groups were introduced to further functionalize the MSN surface with carboxymethyl cellulose (CMC). The amine groups on MSNs covalently react with the –COOH groups of CMC to form amide linkages in the presence of *N*-hydroxy succinimide (NHS).

**Figure 1:**
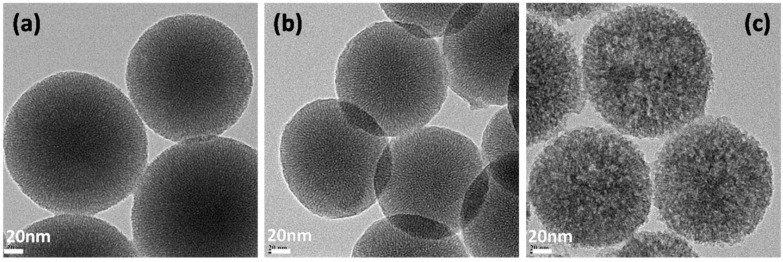
Transmission electron microscopy (TEM) images of (**a**) MSN; (**b**) MSN-NH_2_ and (**c**) MSN-CMC.

TEM images of as-prepared MSNs show uniform discrete spherical nanoparticles with particle sizes in the diameter range of MSN (120 ± 20 nm), MSN-NH_2_ (120 ± 10 nm) and MSN-CMC (120 ± 10 nm). The images also show that MSNs and amine-functionalized MSNs have a porous structure under high magnification (Figure 1a,b). However, this porous structure is covered upon subsequent CMC grafting on to the surface of MSNs (Figure 1c). The observed particle size is in agreement with the sizes obtained from Scanning Electron Microscopy (SEM) (Figure S1 in Supplementary Materials).

**Figure 2:**
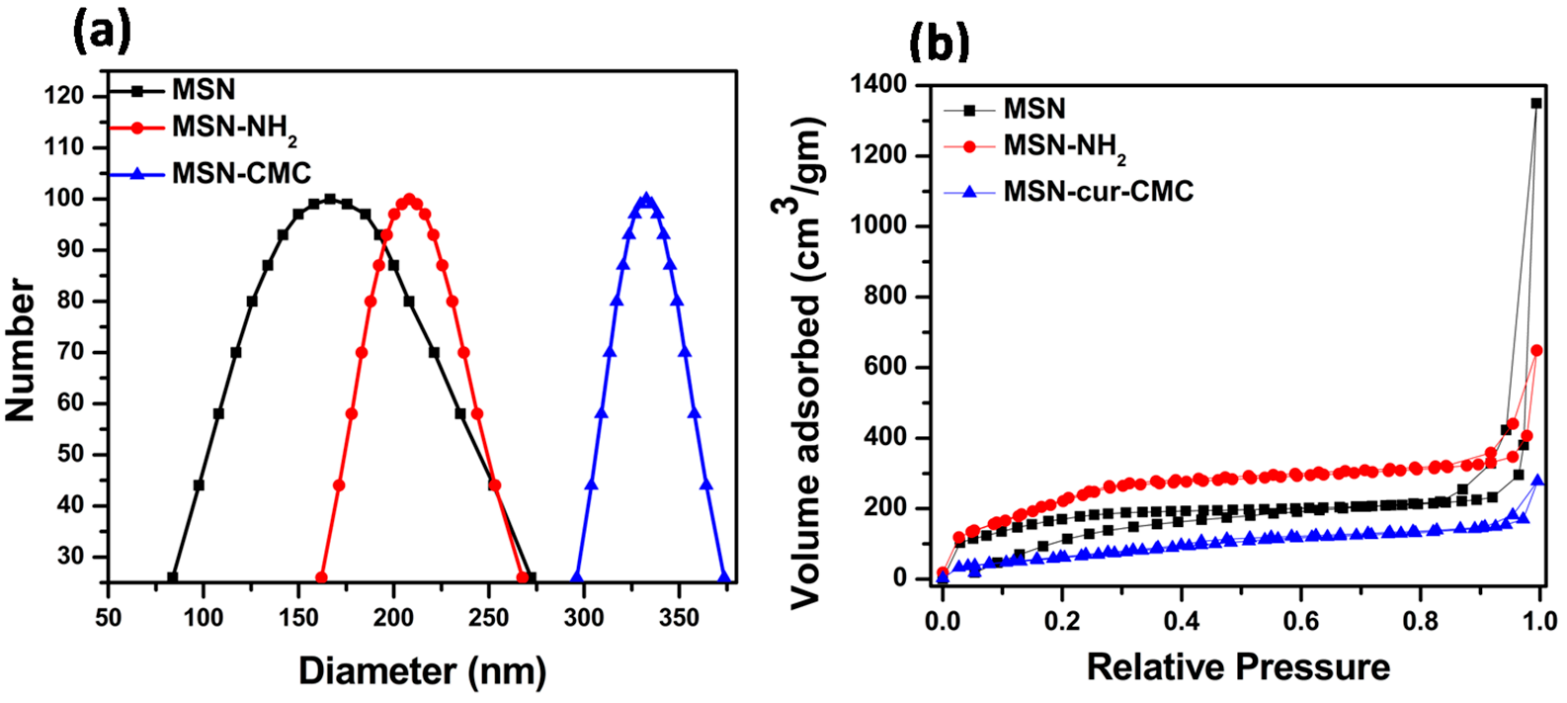
(**a**) Particle size distribution of MSN and functionalized MSNs obtained by DLS experiments and (**b**) nitrogen adsorption-desorption isotherms of MSN, MSN-NH_2_ and MSN-cur-CMC nanoparticles.

Further, the particle size distribution and multimodal size distribution of MSNs with and without functionalization was determined using dynamic light scattering (DLS) experiments (Figures 2a and S2). As shown in the Table 1, the diameters of MSNs were found to be larger as compared to diameters observed from TEM which could be due to the presence of a hydrated layer in the aqueous environment. The MSN-CMC showed a larger diameter of ~333 nm since CMC undergoes gelation and swelling in aqueous medium.

Aqueous electrophoresis experiments were performed on MSNs to determine their ζ potential. It was observed that ζ potential changed from a negative to a positive value upon amine functionalization and later changed to negative value after CMC grafting. This clearly indicated the reaction between the –NH_2_ groups on MSNs and the –COOH groups of CMC (Table 1).

**Figure 3:**
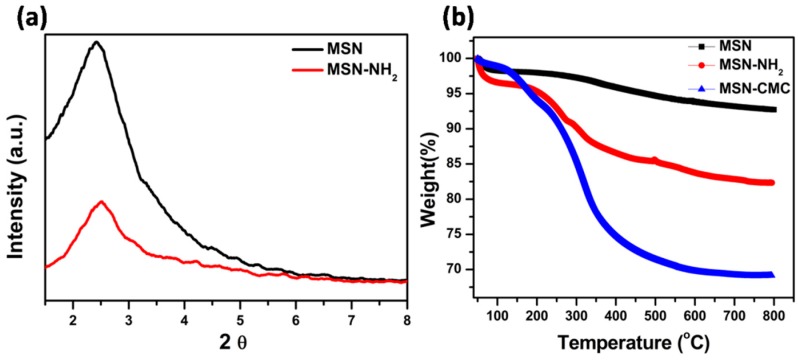
(**a**) X-Ray Diffraction (XRD) patterns of MSN and MSN-NH_2_ and (**b**) Thermogravimetric Analysis (TGA) curves of MSN, MSN-NH_2_ and MSN-CMC.

Nitrogen adsorption desorption isotherms of MSN, MSN-NH_2_ and MSN-cur-CMC showed type IV isotherms, which indicated the mesoporous nature of MSNs as shown in Figure 2b. Barrett, Joyner and Halenda (BJH) method was used for the pore size analysis [35] (see Figure S3). MSNs showed a pore size of ~3 nm, which decreased to 2.7 nm on amine functionalization. This small decrease in the pore size could be due to the presence of certain amine groups inside the pores. The pore size was further reduced to 2 nm in the case of MSN-cur-CMC as a result of successful incorporation of curcumin inside the pores of MSNs. The curcumin incorporation also resulted in a decrease of surface area and the pore volume of MSNs as shown in Table 2. The XRD studies of MSNs and MSN-NH_2_ showed a well resolved diffraction peak at 2Ɵ of 2.44 assigned as 100 plane. This confirms the mesoporous structure of MSNs (Figure 3a).

**Figure 4:**
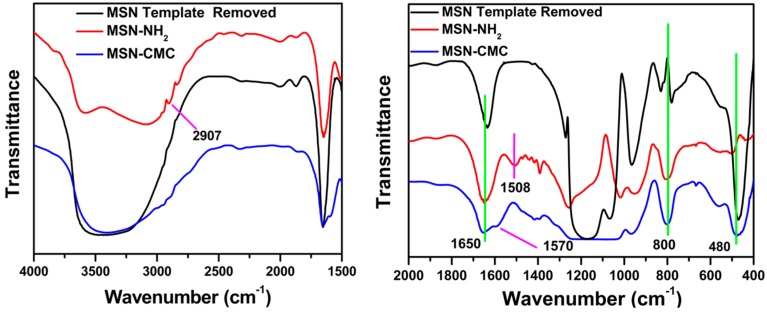
Fourirer Transform Infrared Spectroscopy (FT-IR) Spectra of MSN, MSN-NH_2_ and MSN-CMC.

Further confirmation of the functionalization of MSNs with amine groups and CMC was indicated in the TGA analysis. Figure 3b shows the percentage mass loss profiles as a function of temperature for MSN-NH_2_ and MSN-CMC. It can be seen that after heating the samples up to 800 °C, MSNs, MSN-NH_2_ and MSN-CMC show a mass loss of ~7.3%, 17.5% and 30.7% respectively (Table 1). From these results, the percentage grafting of CMC onto MSNs was calculated to be ~13.2%. We also show in Figure 4, the FT-IR spectra of MSNs, MSN-NH_2_ and MSN-CMC. All the samples showed characteristic peaks at 1650 and 800 cm^−1^ due to Si–O stretching and at 480 cm^−1^ due to Si–O–Si bending. The broad peak at 3400–3500 cm^−1^ corresponds to –OH groups in the samples. MSN-NH_2_ showed a peak at 2907 cm^−1^ due to the C–H stretching of the poly-amino groups and 1508 cm^−1^ due to the –NH bending. However, the peak at 1508 cm^−1^ disappeared in MSN-CMC due to the functionalization of amine groups with CMC. Further, an additional peak appears in MSN-CMC at 1570 cm^−1^ due to the amide bending which is absent in MSN-NH_2_. This further confirms the successful functionalization of MSNs surface with CMC.

### 2.2. Synthesis of Curcumin-Loaded MSNs-Carboxymethyl Cellulose (CMC) Nanoparticles

**Scheme 2:**
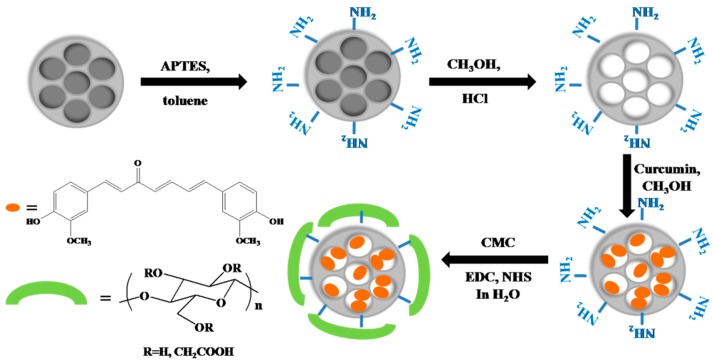
Synthesis of curcumin-loaded carboxymethyl cellulose grafted MSN.

Curcumin, an anticancer drug was effectively loaded into the pores of MSNs in order to increase its bioavailability (since it is hydrophobic in nature) and also to prevent its enzymatic degradation before reaching the cancer cells by EPR effect. For curcumin incubation, MSNs surface was first grafted with amine moieties using APTES. Curcumin was then physically incubated into the pores of MSN-NH_2_ by stirring MSN-NH_2_ in methanol containing curcumin overnight. The curcumin adsorbed on the surface was removed by washing with excess water. The incorporation of curcumin inside the pores of MSNs was confirmed from the reduction in the pore diameter of MSNs from N_2_ adsorption-desorption isotherms (Table 2). In the next step, CMC was grafted onto the surface of curcumin-loaded MSN-NH_2_ by EDC coupling reaction between –NH_2_ groups of MSN-NH_2_ and –COOH groups of CMC. The unreacted reactants and side products if any were removed by washing with distilled water. The synthetic pathway for the curcumin incorporation in MSN-CMC is shown in Scheme 2.

In order to calculate the amount of curcumin loaded into MSN-NH_2_ and MSN-CMC, 1 mg of each material was dispersed in methanol and sonicated for 20 min using probe sonicator. Nanoparticles were then centrifuged and UV absorbance of the supernatant was carried out at 430 nm. The amount of drug loaded in the MSN nanoparticles is given in Table 3. The drug loading content and the drug entrapment efficiency were calculated using the following equations [24]:

(1) $$\mathrm{Drug}\;\mathrm{loading}\;\mathrm{content}\;\left(\%\right)=\frac{\mathrm{Weight}\;\mathrm{of}\;\mathrm{curcumin}\;\mathrm{in}\;\mathrm{MSNs}}{\mathrm{Total}\;\mathrm{weight}\;\mathrm{of}\;\mathrm{loaded}\;\mathrm{MSNs}}\times 100$$

(2) $$\mathrm{Drug}\;\mathrm{Entrapment}\;\mathrm{Efficiency}\;\left(\%\right)=\frac{\mathrm{Weight}\;\mathrm{of}\;\mathrm{curcumin}\;\mathrm{in}\;\mathrm{MSNs}}{\mathrm{Initial}\;\mathrm{weight}\;\mathrm{of}\;\mathrm{curcumin}\;\mathrm{added}}\times 100$$

### 2.3. Release Study of Curcumin from MSN in 0.5% Sodium Lauryl Sulphate

**Figure 5:**
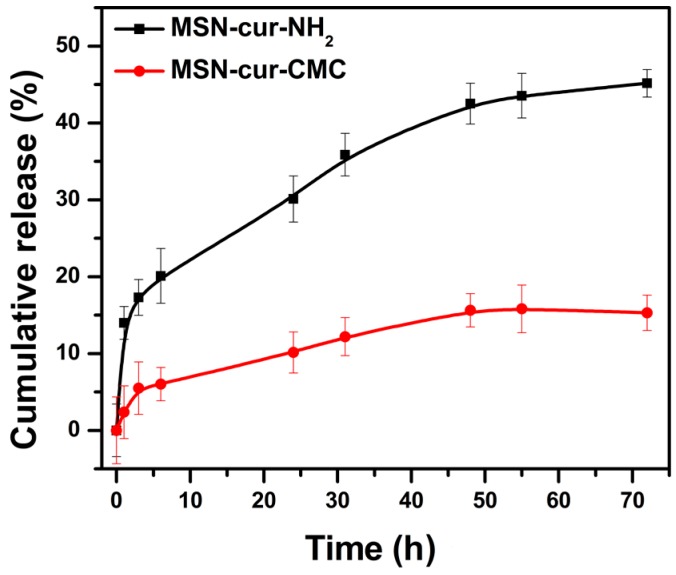
In vitro cumulative release (%) of curcumin from MSN-cur-NH_2_ and MSN-cur-CMC in 0.5% sodium lauryl sulphate (SLS).

The in vitro release of curcumin from MSN-cur-NH_2_ and MSN-cur-CMC was studied by dispersing curcumin loaded MSNs (with same amount of drug loading) in 10 mL 0.5% sodium lauryl sulphate (SLS) solution at 37 °C in a water bath with continuous shaking. In our work, SLS (0.5%) has been used as a drug-releasing medium since curcumin drugs are known to degrade in neutral to basic solutions within a few hours [36]. An aliquot was taken out at fixed time interval and UV absorbance of curcumin at 432 nm was measured. A total of 45% curcumin release was observed in MSN-cur-NH_2_ whereas, only 15% curcumin could be released from MSN-cur-CMC over a period of 72 h (Figure 5). A relatively slower release of curcumin from MSN-cur-CMC could be attributed to the presence of CMC on the surface which creates a barrier for the curcumin molecules to release from the pores of MSN. The slower release of curcumin from MSN-cur-CMC is beneficial since the drug molecules are protected inside the pores of MSNs for a longer period and will be released only after reaching the targeted cancer cells by EPR effect. The drug molecules inside the cells could be easily released by the cleavage of amide linkages (between CMC and MSN-NH_2_) by enzymatic action.

This is indeed observed in the in vitro studies performed on MDA-MB-231 cancer cell lines.

### 2.4. In Vitro Cytotoxicity Assay

**Figure 6:**
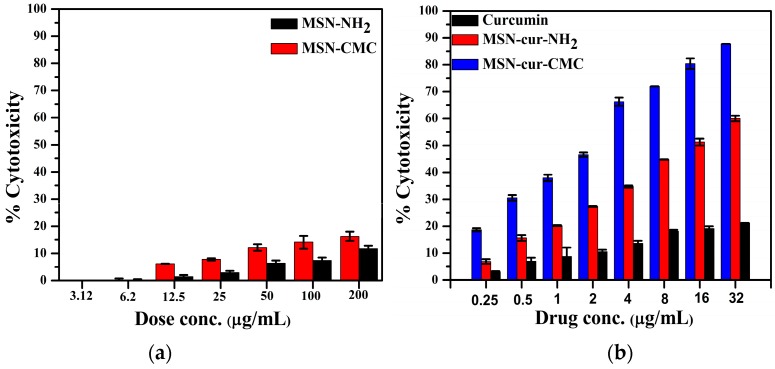
(**a**) % Cytotoxicity of MDAMB 231 cells incubated with MSN-NH_2_ and MSN-CMC and (**b**) % Cytotoxicity of MDA-MB-231 cells incubated with free curcumin, MSN-cur-NH_2_ and MSN-cur-CMC keeping the amount of curcumin same in all the samples (*x* axis represents concentration of free curcumin and curcumin incubated in MSN-NH_2_ and MSN-CMC).

The in vitro cell cytotoxicity of MSN-NH_2_, MSN-CMC, MSN-cur-NH_2_, MSN-cur-CMC and free curcumin to MDA-MB-231 cells was investigated using MTT assay. It can be seen from the Figure 6a that MSN-NH_2_ and MSN-CMC show almost no toxic effect to the cancer cells up to a concentration of 200 µg/mL after incubation for 24 h. The results indicate that MSN-NH_2_ and MSN-CMC are highly biocompatible with the cancer cell line used. Figure 6b showed the in vitro cellular toxicity of MSN-cur-NH_2_, MSN-cur-CMC and free curcumin in MDA-MB-231 cells at different concentrations. It is observed that free curcumin showed negligible cytotoxicity to the cancer cells. This could be due to curcumin being hydrophobic in nature and has very less solubility in the dispersed medium that might affect least contact with the cancer cells. A comparison of the MTT results of MSN-cur-NH_2_ and MSN-cur-CMC with the same concentration of curcumin inside the pores indicated that MSN-cur-CMC has higher cell inhibitory effect as compared to that of MSN-cur-NH_2_. The GI_50_ of MSN-cur-NH_2_ and MSN-cur-CMC are found to be 7 and 1.5 µg/mL respectively (Figure 6b). This clearly indicates that CMC functionalization helps with better internalization of curcumin-loaded MSNs, resulting in better inhibition of the cancer cells as compared to MSN-cur-NH_2_.

### 2.5. Intracellular Uptake of MSN Particles

**Figure 7:**
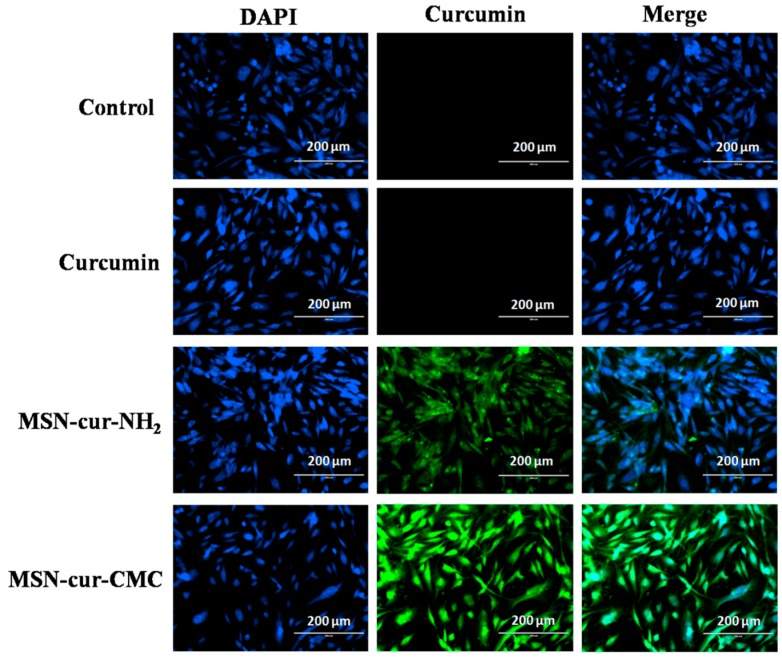
Intracellular uptake of –NH_2_ and –CMC functionalized MSNs using fluorescence microscopy. Images of MDA-MB-231 incubated with 16 µg/mL of free curcumin, MSN-cur-NH_2_ (GI_50_ = 7 µg/mL) and MSN-cur-CMC (GI_50_ = 1.5 µg/mL). Control refers to the non-treated MDA-MB-231 cells. Blue fluorescence is due to nucleus staining of cells with 4’,6-Diamidino-2-Phenylindole Dihydrochloride (DAPI) and green is due to fluorescence of curcumin release inside the cells effectively in MDA-MB-231 cancer cells, which is also in agreement with the MTT assay where the comparable % cytotoxicity in the cells is absent.

For the cellular uptake studies, breast cancer cell line MDA-MB-231 was incubated with free curcumin (16 µg/mL), MSN-cur-NH_2_ and MSN-cur-CMC (GI_50_ conc. of ~7 and 1.5 µg/mL respectively). The GI_50_ concentrations were selected to ensure that the same amount of drug enters the cells (as calculated from MTT assay). Similarly, MSNs without curcumin (MSN-NH_2_ and MSN-CMC) were used as a control at a concentration of 200 µg/mL. The fluorescence of curcumin inside the cells was captured after 1 h of incubation of the MSNs (with and without curcumin) and free curcumin. It was observed that free curcumin does not show fluorescence after 1 h, whereas MSN-cur-NH_2_ and MSN-cur-CMC showed fluorescence of curcumin molecules. It was also evident from the images that MSN-cur-CMC showed much better fluorescence due to curcumin as compared to that of MSN-cur-NH_2_ (Figure 7). This confirms that CMC-grafted MSNs help with better internalization of curcumin inside the cells as compared to MSN-NH_2_ since the GI_50_ of MSN-cur-CMC is much lower compared to that of MSN-cur-NH_2_. The control experiments with MSN-NH_2_ and MSN-CMC do not show any fluorescence, as expected (Figure S4). The results indicate that the CMC-coated MSNs are internalized.

### 2.6. Apoptosis by Fluorescein Isothiocyanate (FITC)-Labeled Annexin V (Annexin V-FITC)/Propidium Iodide (PI) Staining

**Figure 8:**
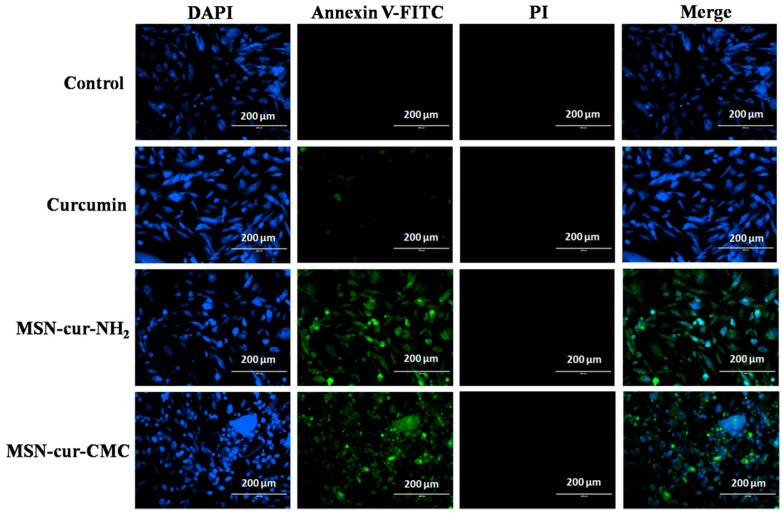
Apoptosis of MDA-MB-231 cells using fluorescence microscopy. Images of MDA-MB-231 incubated with 16 µg/mL of free curcumin, MSN-cur-NH2 (GI_50_ = 7 µg/mL) and MSN-cur-CMC (GI_50_ = 1.5 µg/mL). Control refers to the non-treated MDA-MB-231 cells. Blue fluorescence is due to nucleus staining of cells with DAPI and green fluorescence is due to staining of cells by annexin V-FITC.

**Figure 9:**
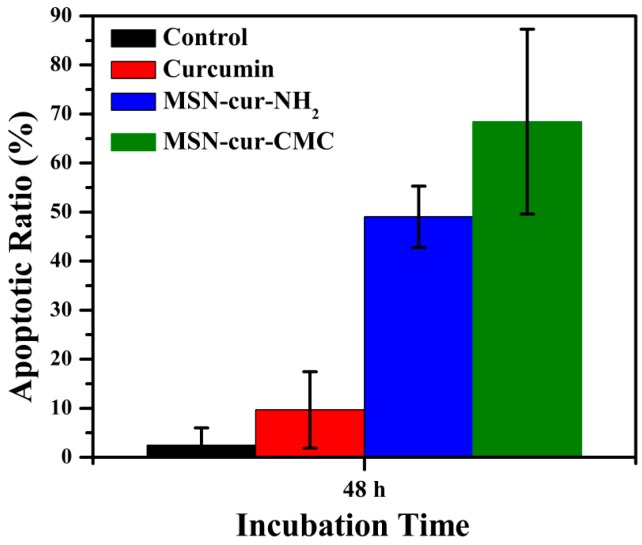
Apoptotic ratios of free curcumin, MSN-cur-NH_2_ and MSN-cur-CMC in 48 h.

In order to find out whether the curcumin-loaded MSNs mediate decreases in cell growth due to apoptosis, we investigated apoptosis in MDA-MB-231 cells using annexin fluorescein isothiocyanate (FITC)-labeled annexin V (annexin V-FITC)/propidium iodide (PI) and 4’,6-Diamidino-2-Phenylindole Dihydrochloride (DAPI) as the staining agents. We observed that treatment of cells with free curcumin (16 µg/mL) and GI_50_ concentrations of MSN-cur-NH_2_ (7 µg/mL) and MSN-cur-CMC (1.5 µg/mL) resulted in increase in the apoptotic cells in 48 h (Figure 8). The MSNs without curcumin, taken as a control, did not show any green fluorescence due to annexin V-FITC/PI, indicating no apoptosis in the cells, and are thus biocompatible (Figure S5). The calculated apoptotic ratios in 48 h for control, curcumin, MSN-cur-NH_2_ and MSN-cur-CMC were found to be 2.5%, 9.7%, 49% and 69% respectively (Figure 9). The high apoptotic value of MSN-cur-CMC compared to free curcumin and MSN-cur-NH_2_ further confirmed that CMC coating on the MSN surface helps with better uptake of MSNs inside the cells and hence more curcumin molecules are released effectively at the targeted site. We also observed that PI positive cells were not observed in the experiment suggesting the absence of necrosis. In order to prove that the green fluorescence observed in the cells after treatment with MSN-cur-NH_2_ and MSN-cur-CMC is due to staining of the cells with annexin V-FITC and not due to fluorescence due to curcumin molecules, we performed the imaging of the cells without annexin V-FITC after 48 h (Figure S6). We observed that in absence of annexin V-FITC, green fluorescence was absent in the cells as the fluorescence due to curcumin do not last for longer time due to quenching (i.e., degradation of curcumin) [36]. This experiment proves that MSN-cur-CMC induces cell death in the MDA-MB-231 cell line via apoptotic pathway.

## 3. Conclusions

In conclusion, we have successfully synthesized MSNs with particle sizes in the range of 120–130 nm and pore size of 2–3 nm as confirmed by TEM, SEM and N_2_ adsorption–desorption studies. The grafting of MSNs with –NH_2_ and CMC moieties on the surface was confirmed by a decrease in the surface area by N_2_– adsorption data and weight loss by TGA data. Also, the zeta potential showed a change in the sign of the charge from positive to negative upon grafting with –NH_2_ and CMC, which also confirmed the functionalization. The drug release profile in 0.5% SLS solution showed only a 15% release of curcumin molecules from MSN-cur-CMC as compared to 45% from MSN-cur-NH_2_ over a period of 72 h. The release profile indicated that CMC helped in preventing curcumin molecules from experiencing premature release over a longer period of time. An MTT assay showed there were negligible toxic effects of MSN-NH_2_ and MSN-CMC on breast cancer cell line MDA-MB-231 up to a concentration of 200 µg/mL, thus indicating biocompatibility of the functionalized MSNs. MSN-cur-CMC showed enhanced cellular uptake and percent cytotoxicity as compared to MSN-cur-NH2 as observed in the MTT assay (from GI_50_ values) on MDA-MB-231 breast cancer cell line. Apoptosis studies performed over a period of 48 h showed that MSN-cur-CMC induces cell death in the MDA-MB-231 cell line via apoptotic pathway. All the above-mentioned observations indicate that CMC grafting on the surface of MSNs enhanced the cellular uptake and cytotoxicity of the cells at remarkably lower concentrations of the curcumin drug. These studies demonstrate that MSN-CMC nanogels could be used as model systems for enhanced cellular uptake and drug delivery applications.

## 4. Materials and Synthesis

### 4.1. Materials

Tetraethylorthosilicate (TEOS) (99%), (3-aminopropyl) triethoxysilane (APTES), hexadecyltrimethylammonium bromide (CTAB) (99%), 1-ethyl-3-(3-dimethylaminopropyl)-carbodiimide hydrochloride (EDC.HCl), *N*-hydroxy succinimide (NHS), sodium lauryl sulphate (SLS), Potassium Bromide (KBr), Polytetrafluoroethylene (PTFE) filters and carboxymethyl cellulose (CMC) were obtained from Sigma Aldrich, St. Louis, MO, USA. Curcumin was a gift sample from Arjuna Natural Extracts, Kerala, India. Dulbecco’s modified Eagle’s medium (DMEM) high glucose, fetal bovine serum (FBS), L-15 medium, Trypsin (0.25% EDTA), annexin-V FITC, 4’,6-Diamidino-2-Phenylindole Dihydrochloride (DAPI), propidium iodide (PI), RNAse A, 4-(2-hydroxyethyl)-1-piperazineethanesulfonic acid (HEPES) buffer, paraformaldehyde (4.7%) and 3-(4,5-dimethylthiazol-2-yl)-2,5-diphenyltetrazolium bromide (MTT) were procured from Invitrogen, Bangalore, India. All chemicals were used as received. The required cell lines for this work (MDA-MB-231) were purchased from National Center for Cell Science (NCCS), Pune, India.

### 4.2. Synthesis

#### 4.2.1. Synthesis of Mesoporous Silica Nanoparticles

One gram of Cetyltrimethylammonium bromide (CTAB) was dissolved in 480 mL of deionized water using an overhead stirrer at room temperature followed by the addition of 2 M NaOH solution (3.5 mL). The solution was allowed to stir for 30 min at 80 °C. 5 mL of tetraethyl orthosilicate (TEOS) was added drop wise to the above mixture. The mixture was stirred at 6000 rpm for another 2 h at 80 °C. The resultant white precipitate was collected by vacuum filtration and washed with copious amount of water. The precipitate was dried in vacuum oven overnight to obtain mesoporous silica in powder form. The removal of the surfactant was carried out by dispersing 1 g of MSN in 100 mL methanol and 1 mL of hydrochloric acid. The solution was refluxed for 6 h. The CTAB removed MSNs were obtained by vacuum filtration and dried in oven overnight.

#### 4.2.2. Outer Surface Functionalization of MSNs with Amino Groups

For the outer surface functionalization of MSNs, 1 g of MSNs (containing CTAB) were dispersed in 100 mL anhydrous toluene followed by addition of 200 µL of 3-aminopropyltriethoxysilane (APTES) in the presence of a catalytic amount of Et_3_N. The reaction mixture was refluxed for 18 h under argon atmosphere. The material was then vacuum filtered, washed with toluene and finally with ethanol. The template (CTAB) was removed by refluxing the material with acidic methanol solution for 6 h. The amine grafted and template removed MSNs were finally washed with methanol and vacuum dried. The obtained material was denoted as MSN-NH_2_.

#### 4.2.3. Curcumin Loading in MSN-NH_2_

To load curcumin into MSN-NH_2_, 200 mg of MSN-NH2 was dispersed in 20 mL of methanol using probe sonicator. To this dispersion, 30 mg of curcumin dissolved in 10 mL methanol was added. The dispersion was stirred overnight at 25 °C. Methanol was then evaporated under vacuum at 50 °C. The curcumin loaded MSNs were then dispersed in water using a sonicator. The MSNs were centrifuged at 12,000 rpm for 10 min and washed several times with water in order to remove any curcumin adsorbed on the surface. The particles were then dried under vacuum overnight to obtain curcumin loaded MSNs. The obtained material was denoted as MSN-cur-NH_2_.

#### 4.2.4. Functionalization of MSN-NH_2_ and MSN-cur-NH_2_ by Carboxymethyl Cellulose

One hundred milligram of MSN-NH_2_ and MSN-cur-NH_2_ were dispersed in 100 mL deionized water using sonication. To this, a premixed solution of NHS (0.4 g), EDC (0.2 g) and carboxymethyl cellulose (150 mg) in 20 mL deionized water was added [37]. The pH of the solution was then adjusted to 9 with an addition of triethylamine. The solution was stirred overnight at 40 °C. The product was centrifuged at 12,000 rpm for 10 min followed by 3 times washing with deionized water to remove any unreacted reactants. The precipitate was then dried under vacuum overnight to obtain curcumin loaded CMC grafted MSN particles. The material was denoted as MSN-cur-CMC (with curcumin) and MSN-CMC (without curcumin).

#### 4.2.5. In Vitro Curcumin Release Studies

The in vitro release of curcumin from MSN-cur-NH_2_ and MSN-cur-CMC were performed in 0.5% SLS solution. Both MSN-cur-NH_2_ and MSN-cur-CMC equivalent to 1 mg of curcumin were suspended in 10 mL 0.5% SLS solution and kept at shaking water bath preset at 37 °C. One milliliter of the supernatant was collected at predetermined time intervals and immediately replaced with equal amount of dissolution medium in order to maintain the sink conditions. The supernatant collected was centrifuged to remove any solid particles and appropriately diluted before taking the absorbance at 432 nm using a UltraViolet-Visible (UV-Vis) spectrophotometer (Shimadzu, Kyoto, Japan). The release profiles of curcumin from MSN-cur-NH_2_ and MSN-cur-CMC were evaluated at 37 °C for 72 h.

#### 4.2.6. In Vitro Cytotoxicity Assay

The sensitivity of MDA-MB-231 cells to the curcumin loaded MSNs was determined by MTT dye uptake as described previously. Briefly, 1 × 10^5^ cells/mL was seeded in a flat bottomed 96-well plate. Next day, the cells were treated with increasing concentrations of blank (3.125–200 µg/mL final concentrations) and curcumin loaded MSNs (0.12–16 µg/mL final drug concentrations) in sterilized MilliQ water and incubated at 37 °C with 5% CO_2_ for 24 h. An untreated group was kept as a negative control and cells treated with free curcumin (0.12–16 µg/mL) were used as positive control. Wells containing culture medium and MTT but no cells acted as blank. After incubation, the MTT solution (5 mg/mL solution in PBS) was added to each well and the cells were incubated for another 3.5 h at 37 °C in 5% CO_2_ incubator. The formazan crystals formed were dissolved by addition of 200 µL of 0.04 N acidified isopropanol. After 15 min, the amount of colored formazan derivative formed was determined by measuring optical density (OD) using the microplate reader at 570 nm. All experiments were conducted in triplicates and the results were presented as average with ±standard deviation. The percentage inhibition was calculated as:

(3) $$\%\;\mathrm{Inhibition}=100-\left[\frac{\left(\mathrm{OD}\;\mathrm{of}\;\mathrm{control}\;\mathrm{well}-\mathrm{OD}\;\mathrm{of}\;\mathrm{treated}\;\mathrm{well}\right)}{\left(\mathrm{OD}\;\mathrm{of}\;\mathrm{control}\;\mathrm{well}-\mathrm{OD}\;\mathrm{of}\;\mathrm{blank}\right)}\times 100\right]$$

#### 4.2.7. Intracellular Uptake of MSNs

The fluorescence of curcumin was used to determine the uptake of curcumin loaded MSNs inside the breast cancer cell line MDA-MB-231. Briefly, 1 × 10^5^ cells/mL was seeded in a glass bottomed black 96-well plate. Next day, cells were treated with MSN-NH_2_ and MSN-CMC (200 µg/mL), curcumin (16 µg/mL) and MSN-cur-NH_2_ and MSN-cur-CMC (GI_50_ = 7 and 1.5 µg/mL final drug concentrations respectively) in sterilized MilliQ water (Merck, Darmstadt, Germany) and incubated at 37 °C with 5% CO_2_ for 1 h. Following incubation, the cells were washed with PBS and stained with DAPI. An alteration in released curcumin level was detected using Laser Scanning Confocal Microscope (LSCM) (Thermo Fischer, Waltham, MA, USA) by measuring the green fluorescence (excitation 490 nm, emission 530 nm).

#### 4.2.8. Apoptosis by Annexin V-FITC/PI Staining

Apoptosis was evaluated by the binding of annexin V-FITC to phosphatidylserine that gets externalized to the outer leaflet of the plasma membrane, followed by high content screening. After 48 h of incubation of the cells with MSN-NH_2_ and MSN-CMC (200 µg/mL), curcumin (16 µg/mL) and MSN-cur-NH_2_ and MSN-cur-CMC (GI_50_ = 7 and 1.5 µg/mL final drug concentrations respectively), the cells were harvested and subsequently treated with annexin V-binding buffer comprising annexin V-FITC (3 µg/mL), DAPI (1 µM) and propidium iodide (10 µg/mL) [38]. The number of cells undergoing apoptosis were examined using LSCM (20× magnification, Olympus FV1000) (Olympus, Melville, NY, USA and Thermo Scientific™ HCS studio™ 2.0 software (Thermo Fischer Scientific, Waltham, MA, USA) was used for three-dimensional multichannel-image processing. The apoptotic ratio was calculated as:

(4) $$\%\;\mathrm{Apoptotic}\;\mathrm{ratio}\;=\frac{\left(\mathrm{Number}\;\mathrm{of}\;\mathrm{cells}\;\mathrm{positive}\;\mathrm{for}\;\mathrm{annexin}\;V-\mathrm{FITC}\right)}{\left(\mathrm{Number}\;\mathrm{of}\;\mathrm{cells}\;\mathrm{positive}\;\mathrm{for}\;\mathrm{DAPI}\right)}\times 100$$

## 5. Characterizations

### 5.1. Fourier Transform Infrared Spectroscopy (FT-IR)

FT-IR spectra were recorded on Perkin Elmer FT-IR spectrum GX instrument (Perkin Elmer, Waltham, MA, USA) using KBr pellets. Pellets were prepared by mixing 3 mg of sample with 97 mg of KBr.

### 5.2. Thermo Gravimetric Analysis

Thermo gravimetric analysis (TGA) of the MSNs were carried out using a TA Instrument SDT Q600 analyzer (TA Instruments, New Castle, DE, USA) between 50 and 800 °C in air (flow 50 mL·min^−1^) at an heating rate of 10 °C·min^−1^. All samples were dried under vacuum at 60 °C overnight prior to TGA runs. The graft density of the grafted moiety on the silica surface was determined by thermo gravimetric analysis (TGA) as described before.

### 5.3. Transmission Electron Microscopy (TEM)

HR-TEM images were taken on a FEI Technai F30 operating (FEI, Hillsboro, OR, USA) at 300 kV with Field Emission Gun (FEG). The samples were prepared by dispersing a 0.1 mg/mL of MSNs in methanol by sonication, dropping the resulting suspension on a copper grid of 400 meshes for 30 s and allowing it to dry in air.

### 5.4. Scanning Electron Microscopy (SEM)

Scanning electron Microscopy (SEM) was used to investigate the morphology of the MSNs using Quanta 200 3D (FEI) dual beam having electron source of tungsten (W) filament with emission at resolution of 20 kV in high vacuum. All the samples were sputter-coated with a thin layer of gold.

### 5.5. Nitrogen Adsorption/Desorption

Nitrogen adsorption/desorption studies at −196 °C were carried out using Quadrasorb *SI* instrument (Quantachrome Instruments, Burlington, ON, Canada). The samples were degassed overnight under vacuum using FloVac Degasser (Quantachrome Instruments, Burlington, ON, Canada) at 100 °C before nitrogen adsorption measurements. Multi-point Brunauer-Emmett-Teller (BET) surface area was obtained from the nitrogen adsorption isotherm in the relative pressure range from 0.1 to 0.3. Pore sizes were calculated using the Barrett, Joyner and Halenda (BJH) method from adsorption branch of the isotherm in the relative pressure range from 0.3 to 0.99 units and total pore volume was calculated at *P*/*P*_o_ of 0.99.

### 5.6. ζ Potential and Size Determination

The hydrodynamic diameters of dilute aqueous solutions of the MSNs, MSN-NH_2_ and MSN-CMC were determined by dynamic light scattering (DLS) (Brookhaven Instruments, Holtsville, NY, USA) equipped with a He–Ne laser operating at 632 nm. The particle size was calculated using 90 Plus particle Sizing Software Ver. 3.94 (Brookhaven Instruments). Sample solutions 1 mg/mL were made in water and were filtered using a 0.8 µm Polytetrafluoroethylene (PTFE) filter.

Aqueous electrophoretic data for the above mentioned MSNs were obtained using Brookhaven Instruments. For each sample three measurements were taken and the average value is reported. ζ potentials were calculated using PALS ζ Potential Analyzer Software Ver. 3.54 (Brookhaven Instruments).

## Figures and Tables

**Table 1:** Physical properties of various MSNs.

Sample Name	Weight Loss (%)	Hydrodynamic Diameter (nm)	Mean ζ Potential (mV) in Water
MSN	7.3	167 ± 5	−34.2 ± 2
MSN-NH_2_	17.5	210 ± 7	38.48 ± 3
MSN-CMC	30.7	333 ± 4	−3.07 ± 2

MSN, mesoporous silica nanoparticles; CMC, carboxymethyl cellulose.

**Table 2:** Nitrogen adsorption-desorption analysis of MSNs.

Sample Name	Surface Area (m^2^/g)	Pore Diameter (nm)	Pore Volume (cm^3^/g)
MSN	939.6	3	0.738
MSN-NH_2_	659.1	2.6	0.642
MSN-cur-CMC	262.9	2	0.339

**Table 3:** Curcumin loading in functionalized MSNs.

Sample Name	Drug Loading Content (%)	Drug Entrapment Efficiency (%)
MSN-cur-NH_2_	10.7	71.3
MSN-cur-CMC	8	53.3

## Supplementary Materials

The following are available online at www.mdpi.com/2310-2861/3/1/8/s1, Figure S1: SEM image of as synthesized MSNs; Figure S2: DLS of MSN, MSN-NH_2_ and MSN-CMC; Figure S3: Pore diameter of MSN, MSN-NH_2_ and MSN-cur-CMC using BJH method from N_2_-adsortion desorption studies; Figure S4: Intracellular uptake of MSN-NH_2_ and MSN-CMC using fluorescence microscopy. Images are at a magnification of 200 µm of MDA-MB-231 incubated with a concentration of 200 µg/mL. Blue fluorescence is due to nuclei staining of cell with DAPI; Figure S5: Apoptosis of MDA-MB-231 cells using fluorescence microscopy. Images are at a magnification of 200 µm of MDA-MB-231 incubated with 200 µg/mL of MSN-NH_2_ and MSN-CMC. Blue fluorescence is due to nuclei staining of cell with DAPI and Figure S6: Intracellular uptake of –NH_2_ and –CMC functionalized MSNs using fluorescence microscopy after 48 h. Images of MDA-MB-231 incubated with 16 µg/mL of free curcumin, MSN-cur-NH_2_ (GI_50_ = 7 µg/mL) and MSN-cur-CMC (GI_50_ = 1.5 µg/mL). Control refers to the non treated MDA-MB-231 cells. Blue fluorescence is due to nuclei staining of cell with DAPI and green due to fluorescence of curcumin release inside the cells.
