# Supplementary material for: Carboxymethyl Cellulose-Grafted Mesoporous Silica Hybrid Nanogels for Enhanced Cellular Uptake and Release of Curcumin
Source: Gels. 2017 Feb 22;3(1):8. doi: 10.3390/gels3010008 (PMC6318674; doi:10.3390/gels3010008)
Supplement: Supplementary file 1 [file gels-03-00008-s001.pdf]

Supporting Information

Carboxymethyl cellulose Grafted Mesoporous Silica hybrid Nanogels for enhanced Cellular uptake and release of Curcumin

Neha Tiwari[1, 3], Laxman Nawale[2], Dhiman Sarkar[2,3] and Manohar V. Badiger[1,3]*
[1] Polymer Science and Engineering Division, CSIR- National Chemical Laboratory, Pune- 411008
[2]Combichem Bioresource Centre, Organic Chemistry Division, CSIR-National Chemical Laboratory, Pune- 411008
[3]Acedamy of Scientific & Innovative Research, CSIR-NCL Campus, Pune-411008

E-mail: mv.badiger@ncl.res.in; Tel: +91-20-25902187; Fax:+91-20-2590-2612

1. **SEM of MSNs**

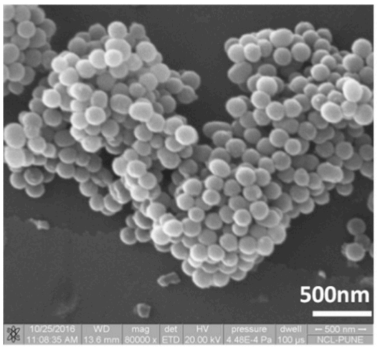

Fig.1S. SEM image of as synthesized MSNs

2. **Multimodal distribution of as synthesized and Functionalized MSNs**

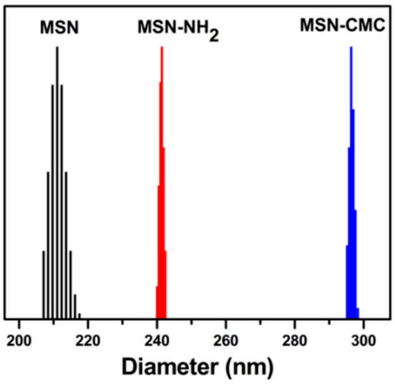

Fig.2S. DLS of MSN, MSN-NH_2 and MSN-CMC

3. Pore diameter of MSN

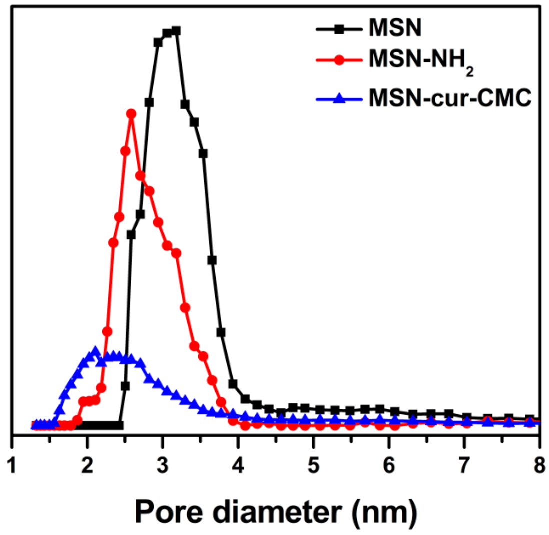

Fig.3S. Pore diameter of MSN, MSN-NH_2 and MSN-cur-CMC using BJH method from N_2-adsortion desorption studies

4. Cellular uptake of MSN-NH_2 and MSN-CMC

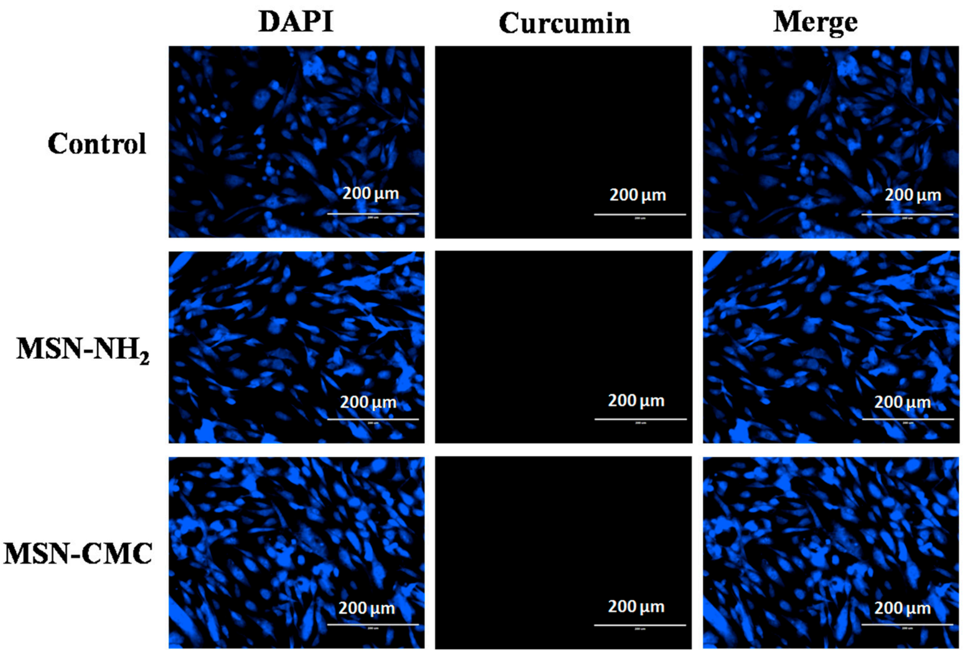

Fig.4S. Intracellular uptake of MSN-NH_2 and MSN-CMC using fluorescence microscopy. Images of MDA-MB-231 incubated with 200µg/ml of MSN-NH_2 and MSN- CMC. Control refers to the non treated MDA-MB-231 cells. Blue fluorescence is due to nuclei staining of cell with DAPI

5. **Apoptosis of $MSN\text{-}NH_2$ and MSN-CMC**

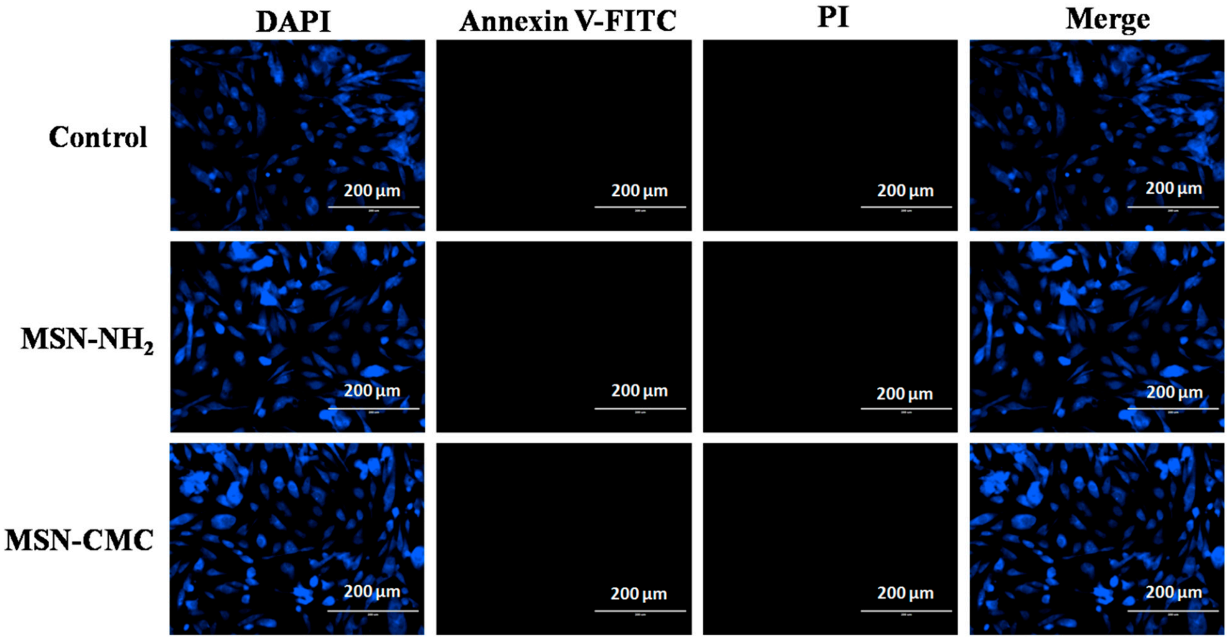

Fig.5S. Apoptosis of MDA-MB-231 cells using fluorescence microscopy. Images are at a magnification of 200µm of MDA-MB-231 incubated with 200µg/ml of $MSN\text{-}NH_2$ and MSN- CMC. Images of MDA-MB-231 incubated with 200µg/ml of $MSN\text{-}NH_2$ and MSN- CMC. Control refers to the non treated MDA-MB-231 cells. Blue fluorescence is due to nuclei staining of cell with DAPI

6. Fluorescence due to curcumin from MSN-cur-NH_2 and MSN-cur-CMC after 48hrs

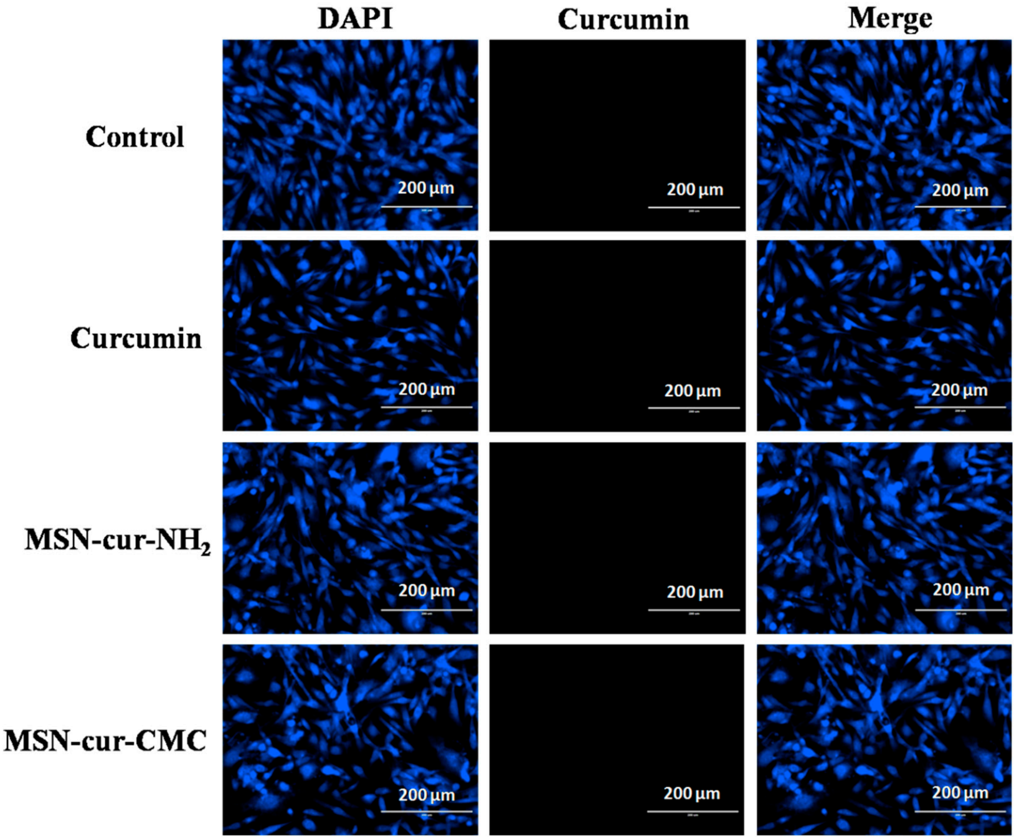

Fig.6S. Intracellular uptake of –NH_2 and –CMC functionalized MSNs using fluorescence microscopy after 48hrs. Images of MDA-MB-231 incubated with 16µg/ml of free curcumin, MSN-cur-NH_2 (GI_{50}=7µg/ml) and MSN-cur-CMC (GI_{50}=1.5µg/ml). Control refers to the non treated MDA-MB-231 cells. Blue fluorescence is due to nuclei staining of cell with DAPI and green due to fluorescence of curcumin release inside the cells.
